# Austin District Medical Society; Annual Meeting and Banquet, Etc.

**Published:** 1888-10

**Authors:** T. J. Bennett

**Affiliations:** Sec’y.


THE AUSTIN DISTRICT MEDICAL SOCIETY held its quarterly meeting in the city of Austin on the 20th of September, ult.

There were thirty-six physicians in attendance and much interest was shown in every subject brought before the Society.

The following names were added to the list of membership: Drs. R. P. Talley, of Belton; A. V. Doak, of Taylor; J. W. Harrison, of Dripping Springs; J. H. McCaleb, of Garfield; W. P. Flemming, of Georgetown and Joseph Cummings, of Austin.

Dr. Hubbard, a prominent medical gentleman of Tennessee, being on a visit to relatives in Austin, was present, by invitation, and took part in the discussions. Also Dr. E. J. Beall, of Fort Worth, who was passing through the city, stopped over, and spent the day with the Society.

The following papers were read and discussed: "Tuberculosis of the Joints," by W. M. Cunningham, of Bastrop; "Puerperal Eclampsia," by Dr. W. P. Flemming, of Georgetown; "Railway Corporations and their Responsibilities to Society," by Dr. R. M. Swearingen; "Dilatation of the Heart," by Dr. John Preston of the Lunatic Asylum; "Insanity; its Nature and Causes," by Dr. A. N. Denton, of Austin.

At 2 o'clock, p. m., the Society enjoyed the annual banquet in a body at Billeisen's Restaurant, after which the Society elected officers for the ensuing year;

Dr. W. A. Morris, of Austin, was elected President; Dr. A. N. Denton, first Vice-President, and R. S. Gregg, of Manor, second Vice-President; Dr. John Preston and Dr. T. O. Maxwell were elected members of the Board of Censors.

On motion the Society adjourned to meet again in Austin in December next. T. J. BENNETT, Sec'y.

Discussions of papers will appear simultaneously with the publication of respective papers in the official organ,—DANIEL'S TEXAS MEDICAL JOURNAL.

